# Supplementary material for: Iron status and anemia in a representative sample of US pregnant women is not associated with pre-pregnancy BMI: Results from the NHANES (1999–2010) study
Source: PLoS One. 2024 Sep 10;19(9):e0300912. doi: 10.1371/journal.pone.0300912 (PMC11386453; doi:10.1371/journal.pone.0300912)
Supplement: S1 File — S1 Table: Weighted medians and prevalences of inflammation, iron, and anemia biomarkers in pregnant women of reproductive age (18–49 years of age) from the NHANES (1999–2010) study (n = 1156). S2 Table: Weighted prevalences and prevalence ratios and differences of iron deficiency and anemia status in pregnant women ages 18–49 by race/ethnicity from NHANES 1999–2010 (n = 1156). S3 Table: Weighted prevalences and prevalence ratios and differences of iron deficiency and anemia status in pregnant women ages 18–49, by race/ethnicity from NHANES 1999–2010 (n = 1156) using Mexican American and Hispanic Other categories. (DOCX) [file pone.0300912.s001.docx]

**Supplementary Data - Iron status in a representative sample of US pregnant women is not associated with pre-pregnancy BMI: results from the NHANES (1999-2010) study**

**S1 Table**: **Weighted medians and prevalences of inflammation, iron, and anemia biomarkers in pregnant women of reproductive age (18-49 years of age) from the NHANES (1999-2010) study (n=1156).**

|  | **Total sample** | |
| --- | --- | --- |
| **Variables** | **N^a^** | **Median [IQR] or % (SE)** |
| **C-reactive protein, mg/L** | 1156 | 4.8 [2.5-8.8] |
| Inflammation (> 5 mg/L) | 605 | 47.8 (2.5) |
| **Ferritin, µg/L** | 1156 | 27.0 [14.0-52.0] |
| ID (< 12 µg/L) | 276 | 19.4 (1.85) |
| **TfR, mg/L** | 1156 | 5.00 [4.25-6.20] |
| ID (> 4.4 mg/L) | 212 | 16.6 (1.68) |
| **Total body iron**^b^**, mg/kg** | 1156 | 4.53 [1.87-7.30] |
| ID (< 0 mg/kg) | 211 | 14.2 (1.38) |
| **Hb, g/dL** | 1156 | 12.4 [11.8-13.1] |
| Anemia (< 11 g/dL) | 100 | 7.55 (1.27) |
| **Iron deficiency anemia** |  |  |
| Ferritin < 12 µg/L and Hb < 11 g/dL | 51 | 3.24 (0.72) |
| TfR > 4.4 mg/L and Hb < 11 g/dL | 48 | 3.06 (0.74) |
| Total body iron < 0 mg/kg and Hb < 11 g/dL | 49 | 2.75 (0.69) |
| ^a^ N values were non-weighted; all other analyses were weighted.  ^b^ Total body iron was calculated using Ferritin and TfR using Cook’s equation [1]  Abbreviations: Hb=Hemoglobin; ID=Iron deficiency; IQR=interquartile range TfR=Transferrin receptor | | |

We ran adjusted prevalence models for ID and anemia by race/ethnicity categories. This is similar to analysis performed by previous researchers but complements it by examining adjusted prevalence ratios [2]. Similar to these previous reports, we found that prevalence of ID and anemia were highest in non-Hispanic Black pregnant women. The prevalence of ID in Hispanic women did not differ from non-Hispanic White women **(S2 Table 2)**. However, non-Hispanic Black women had approximately two times higher adjusted prevalence of ID when compared to non-Hispanic White women, based on cutoffs for serum Ferritin, TfR, and total body iron biomarkers. The prevalence of ID defined by TfR was increased by 18 cases per hundred participants in non-Hispanic Black women compared to non-Hispanic White women. Non-Hispanic Black women also had an adjusted anemia prevalence ratio of 5.6 times higher than non-Hispanic White women. Because the number of cases of ID with anemia was low within each race/ethnicity category, the estimates in adjusted models were considered unreliable and should be interpreted with caution. Similarly, the number of cases for all outcomes was low in the Non-Hispanic Other category.

**S2 Table**: **Weighted prevalences and prevalence ratios and differences of iron deficiency and anemia status in pregnant women ages 18-49 by race/ethnicity from NHANES 1999-2010 (n = 1156).**

| **Race/ethnicity categories by iron status:** | **Prevalence**  **[% (Standard Error)]** | **Cases (N)^a^** | **Unadjusted Prevalence Ratio (95% CI)^b^** | **Adjusted Prevalence Ratio (95% CI)^c^** | **Adjusted Prevalence Difference**  **[excess cases per 100**  **(95% CI)]** |
| --- | --- | --- | --- | --- | --- |
| **ID (Ferritin < 12 ug/L)** | | | | |  |
| Hispanic | 24.3 (2.68) | 115 | 1.59 (1.05, 2.40) | 1.28 (0.83, 1.98) | 4.61 (-3.25, 12.5) |
| Non-Hispanic White | 15.3 (2.48) | 103 | Reference | Reference | Reference |
| Non-Hispanic Black | 29.0 (4.97) | 45 | 1.90 (1.19, 3.02) | 1.67 (1.04, 2.69) | 10.9 (0.00, 21.8) |
| Non-Hispanic Other | 16.6 (7.26) | 13 | 1.09 (0.44, 2.66)^d^ | 1.22 (0.57, 2.59)^d^ | 3.57 (-11.0, 18.2)^d^ |
| **ID (TfR > 4.4 mg/L)** | | | | |  |
| Hispanic | 15.7 (3.11) | 79 | 1.37 (0.80, 2.34) | 1.28 (0.73, 2.24) | 3.26 (-4.35, 10.9) |
| Non-Hispanic White | 11.5 (2.21) | 64 | Reference | Reference | Reference |
| Non-Hispanic Black | 30.1 (4.42) | 53 | 2.62 (1.59, 4.31) | 2.49 (1.43, 4.33) | 17.5 (6.29, 28.7) |
| Non-Hispanic Other | 26.3 (8.44) | 16 | 2.29 (1.07, 4.93)^d^ | 2.29 (1.20, 4.36)^d^ | 15.1 (0.40, 29.8)^d^ |
| **ID (Total body iron < 0 mg/kg)^e^** | | | | |  |
| Hispanic | 17.5 (2.56) | 89 | 1.65 (0.99, 2.75) | 1.29 (0.77, 2.15) | 3.29 (-3.28, 9.86) |
| Non-Hispanic White | 10.6 (1.99) | 72 | Reference | Reference | Reference |
| Non-Hispanic Black | 23.3 (4.30) | 39 | 2.20 (1.31, 3.68) | 1.80 (1.00, 3.23) | 9.15 (-0.66, 19.0) |
| Non-Hispanic Other | 12.7 (6.94) | 11 | 1.20 (0.35, 4.15)^d^ | 1.45 (0.53, 3.97)^d^ | 5.15 (-10.5, 20.8)^d^ |
| **Anemia (Hb < 11 g/dL)^f^** | | | | |  |
| Hispanic | 8.35 (2.05) | 38 | 2.54 (1.03, 6.24) | 2.05 (0.72, 5.88) | 3.65 (-1.65, 8.95) |
| Non-Hispanic White | 3.28 (1.29) | 25 | Reference | Reference | Reference |
| Non-Hispanic Black | 18.5 (3.81) | 30 | 5.62 (2.38, 13.3) | 5.45 (2.23, 13.3) | 15.4 (6.63, 24.3) |
| Non-Hispanic Other | 12.7 (6.01) | 7 | 3.86 (1.11, 13.4)^d^ | 3.89 (1.22, 12.4)^d^ | 10.0 (-3.08, 23.1)^d^ |
| **ID Anemia (Hb < 11 g/dL and Total body iron < 0 mg/kg)^f^** | | | | | |
| Hispanic | 3.07 (0.91) | 21 | 1.85 (0.51, 6.67) | 1.70 (0.49, 5.83) | 1.14 (-1.32, 3.59) |
| Non-Hispanic White | 1.66 (0.93) | 14 | Reference | Reference | Reference |
| Non-Hispanic Black | 7.67 (2.85) | 12 | 4.61 (1.23, 17.23) | 4.70 (1.42, 15.5) | 6.04 (0.37, 11.7) |
| Non-Hispanic Other | 0.64 (0.47) | 2 | 0.38 (0.06, 2.44)^d^ | 0.60 (0.10, 3.50)^d^ | -0.66 (-2.85, 1.53)^d^ |

^a^ N values were non-weighted; all other analyses were weighted.

^b^ Prevalence ratios and prevalence differences were calculated using Poisson regression models with robust error variance, with and without covariates.

^c^ The covariates used in adjusted models were age, BMI, education level, and trimester of pregnancy.

^d^ Results for the non-Hispanic Other race/ethnicity group may be unreliable because the number of cases was < 30 for all models [3].

^e^ Total body iron was calculated using Ferritin and TfR receptor using Cook’s equation [1].

^f^ Estimates may be unreliable due to sample size of cases < 30 for some race categories [3].

Abbreviations: BMI=Body Mass Index; CI=Confidence interval; Ferritin=Ferritin; ID=Iron deficiency; Hb=Hemoglobin; TfR=Transferrin receptor

Because the 1999-2006 NHANES cycles oversampled the Mexican American population, the Hispanic category for these years represents this sub-population only. In order to test how this affected prevalence estimates for ID and anemia, we performed a sensitivity analysis using the Mexican American and Other Hispanic groups. Results for the Mexican American group was the same as the total Hispanic group in adjusted models. The Hispanic Other group was too small to calculate reliable estimates but had similar results to the Mexican American group in adjusted models. One potential exception is that the Hispanic Other group had higher prevalence of ID when determined by TfR > 4.4 mg/L.

**S3 Table**: **Weighted prevalences and prevalence ratios and differences of iron deficiency and anemia status in pregnant women ages 18-49, by race/ethnicity from NHANES 1999-2010 (n=1156) using Mexican American and Hispanic Other categories.**

| **Race/ethnicity categories by iron status:** | **Prevalence**  **[% (Standard Error)]** | **Cases (N)^a^** | **Unadjusted Prevalence Ratio (95% CI)^b^** | **Adjusted Prevalence Ratio (95% CI)^c^** | **Adjusted Prevalence Difference**  **[excess cases per 100**  **(95% CI)]** |
| --- | --- | --- | --- | --- | --- |
| **ID (Ferritin < 12 ug/L)** | | | | |  |
| Mexican American | 28.1 (2.84) | 104 | 1.83 (1.28, 2.63) | 1.49 (0.99, 2.23) | 7.86 (0.08, 15.8) |
| Non-Hispanic White | 15.3 (2.48) | 103 | Reference | Reference | Reference |
| Non-Hispanic Black | 29.0 (4.97) | 45 | 1.90 (1.19, 3.02) | 1.69 (1.05, 2.71) | 11.1 (0.19, 22.1) |
| Non-Hispanic Other | 16.6 (7.26) | 13 | 1.09 (0.44, 2.66)^d^ | 1.21 (0.57, 2.58)^d^ | 3.47 (-11.0, 18.0)^d^ |
| Hispanic Other | 13.2 (6.02) | 11 | 0.86 (0.30, 2.51)^d^ | 0.72 (0.26, 1.96)^d^ | -4.53 (-17.3, 8.20)^d^ |
| **ID (TfR > 4.4 mg/L)** | | | | |  |
| Mexican American | 12.7 (2.47) | 65 | 1.11 (0.65, 1.89) | 1.04 (0.58, 1.86) | 0.46 (-6.55, 7.46) |
| Non-Hispanic White | 11.5 (2.21) | 64 | Reference | Reference | Reference |
| Non-Hispanic Black | 30.1 (4.42) | 53 | 2.62 (1.59, 4.31) | 2.46 (1.41, 4.29) | 17.2 (6.05, 28.4) |
| Non-Hispanic Other | 26.3 (8.44) | 16 | 2.29 (1.07, 4.93)^d^ | 2.30 (1.20, 4.39)^d^ | 15.3 (0.38, 30.2)^d^ |
| Hispanic Other | 24.6 (8.21) | 14 | 2.15 (1.02, 4.51)^d^ | 1.88 (1.00, 3.55)^d^ | 10.4 (-0.21, 22.9)^d^ |
| **ID (Total body iron < 0 mg/kg)^e^** | | | | |  |
| Mexican American | 18.1 (2.38) | 78 | 1.71 (1.10, 2.66) | 1.31 (0.80, 2.16) | 3.57 (-2.88, 10.0) |
| Non-Hispanic White | 10.6 (1.99) | 72 | Reference | Reference | Reference |
| Non-Hispanic Black | 23.3 (4.30) | 39 | 2.20 (1.31, 3.68) | 1.80 (1.01, 3.23) | 9.17 (-0. 65, 19.0) |
| Non-Hispanic Other | 12.7 (6.94) | 11 | 1.20 (0.35, 4.15)^d^ | 1.45 (0.53, 3.97)^d^ | 5.14 (-10.5, 20.8)^d^ |
| Hispanic Other | 15.7 (7.13) | 11 | 1.48 (0.51, 4.35)^d^ | 1.21 (0.50, 2.94)^d^ | 2.45 (-9.21, 14.1)^d^ |
| **Anemia (Hb < 11 g/dL)^f^** | | | | |  |
| Mexican American | 7.64 (1.80) | 30 | 2.33 (0.94, 5.73) | 1.88 (0.63, 5.54) | 3.04 (-2.17, 8.25) |
| Non-Hispanic White | 3.28 (1.29) | 25 | Reference | Reference | Reference |
| Non-Hispanic Black | 18.5 (3.81) | 30 | 5.62 (2.38, 13.3) | 5.42 (2.21, 13.3) | 15.4 (6.54, 24.2) |
| Non-Hispanic Other | 12.7 (6.01) | 7 | 3.86 (1.11, 13.4)^d^ | 3.90 (1.22, 12.4)^d^ | 10.1 (-3.10, 23.3)^d^ |
| Hispanic Other | 10.4 (5.36) | 8 | 3.18 (0.91, 11.1)^d^ | 2.51 (0.69, 9.18)^d^ | 5.26 (-4.03, 14.5)^d^ |
| **ID Anemia (Hb < 11 g/dL and Total body iron < 0 mg/kg)^f^** | | | | | |
| Mexican American | 3.55 (1.21) | 18 | 2.13 (0.57, 8.02) | 1.93 (0.53, 7.01) | 1.51 (-1.38, 4.40) |
| Non-Hispanic White | 1.66 (0.93) | 14 | Reference | Reference | Reference |
| Non-Hispanic Black | 7.67 (2.85) | 12 | 4.61 (1.23, 17.2) | 4.71 (1.42, 15.6) | 6.05 (0.39, 11.7) |
| Non-Hispanic Other | 0.64 (0.47) | 2 | 0.38 (0.06, 2.44)^d^ | 0.59 (0.10, 3.47)^d^ | -0.67 (-2.85, 1.51)^d^ |
| Hispanic Other | 1.67 (0.92) | 3 | 1.00 (0.20, 4.98)^d^ | 0.98 (0.21, 4.50)^d^ | 0.03 (-2.50, 2.43)^d^ |

^a^ N values were non-weighted; all other analyses were weighted.

^b^ Prevalence ratios and prevalence differences were calculated using Poisson regression models with robust error variance, with and without covariates.

^c^ The covariates used in adjusted models were maternal age, maternal BMI, maternal education level, and trimester of pregnancy.

^d^ Results for the non-Hispanic Other and Hispanic Other race/ethnicity groups may be unreliable because the number of cases was < 30 for all models [3].

^e^ Total body iron was calculated using Ferritin and TfR receptor using Cook’s equation [1].

^f^ Estimates may be unreliable due to sample size of cases < 30 for some race categories [3].

Abbreviations: BMI=Body Mass Index; CI=Confidence interval; ID=Iron deficiency; Hb=Hemoglobin; TfR=Transferrin receptor

**References**

1. Cook JD, Flowers CH, Skikne BS. The quantitative assessment of body iron. Blood. 2003;101: 3359–3363. doi:10.1182/blood-2002-10-3071

2. Mei Z, Cogswell ME, Looker AC, Pfeiffer CM, Cusick SE, Lacher DA, et al. Assessment of iron status in US pregnant women from the National Health and Nutrition Examination Survey (NHANES), 1999-2006. Am J Clin Nutr. 2011;93: 1312–1320. doi:10.3945/ajcn.110.007195

3. Parker JD, Talih M, Malec DJ, Beresovsky V, Carroll M, Gonzalez JF, et al. National Center for Health Statistics Data Presentation Standards for Proportions. Vital Health Stat 2. 2017; 1–22.
