# Supplementary material for: Iron status and anemia in a representative sample of US pregnant women is not associated with pre-pregnancy BMI: Results from the NHANES (1999–2010) study
Source: PLoS One. 2024 Sep 10;19(9):e0300912. doi: 10.1371/journal.pone.0300912 (PMC11386453; doi:10.1371/journal.pone.0300912)
Supplement: Attachment — Submitted filename: Response to reviewers_May.9.2024.docx [file pone.0300912.s002.docx]

**Thank you to the editors and reviewers for their careful consideration of our manuscript. Below are our responses to the comments from the editors and reviewers. Author responses are below editor and reviewer comments in blue. All line numbers refer to the clean manuscript version.**

In addition to reviewers´ comments, please describe the procedures for selection of covariables used in the multiple adjusted models.

Author comments: We selected covariables *a priori* based on prior literature of factors known to be related to iron and anemia status and BMI during pregnancy (please see the Methods/Covariates section, lines 136-140).

One important concern is the small number of observations for the outcomes (anemia and iron deficiency anemia): this point should be discussed in relation to the limitations of sample power and external validity.

Author comments: Thank you for pointing out the small number of observations for the anemia and iron deficiency anemia outcomes. We followed the CDC guidelines and flagged the estimates that had a small sample size (<30). We thought the readers should see these results but interpret them with caution given the small sample size. We pointed this limitation out in the table footnotes, Results section (lines #237-240) and Strengths & Limitations section (lines #357-360).

Reviewer #2 also had questions regarding our sample size and power, please see our answer under Methods (first question).

**Comments to the Author**

**Reviewer #1:** Thank you for the opportunity to read this interesting manuscript. I am pleased to see this paper that explores the association between pre-pregnancy BMI, iron status and anemia in a nationally representative sample of US pregnant women.
In my opinion, the manuscript is a good piece of work, well structured, clear and easy to follow and understand. The introduction section is well presented, provides a contextualization of the topic addressed and presents the justification for the study. The methods section provides sufficient information, is well detailed and can be replicated. The statistical analyses are sound and the results are robust. The conclusions are well-stated and, in the discussion section, the authors explain the results and make relevant comparisons with previous studies.

I have only 2 minor suggestions:
1. I suggest to include anemia in the title and objective of the study, since you also assessed this outcome in addition to iron status.

Author comments: Thank you for this helpful suggestion, which will provide more clarity to our readers regarding what we examined. We incorporated the term anemia in our title (lines 1-2), objective (line 75), and throughout the manuscript.

2. In the abstract (line 36), check the PR value for ID (overweight). It is different from what is shown in Table 2.

Author comments: Thanks for spotting this discrepancy; it is now fixed. Line 37: “There were no differences in the prevalence of ID or anemia in women with pre-pregnancy overweight and obesity (ID: overweight, adjusted prevalence ratio (PR)=1.27, 95%CI: 0.89-1.82; obesity, PR=0.75, 95%CI: 0.39-1.45.”

In addition, we found that the p-values reported in the Figure 1 legend did not match the figure because they were from a previous version of the statistical analysis. We have updated the legend to be the correct numbers (lines 228-229).

**Reviewer #2**: Reviewer Report:
This is an interesting study on Iron status among US pregnant women.

I propose to address the following minor issues.

Abstract:
Abstract was written properly.

Introduction:
Introduction is written properly.

Methods:
Study population: How the sample size was calculated? Is this sample sufficient to produce enough power?

Author comments: Thanks for this question. As described in lines 81-91, our sample size was based on years of available NHANES data in which iron status biomarkers were measured. Importantly, our sample size of 1,156 was larger than other US studies that have investigated the association between BMI and iron status (Cao et al., 2016 and Dao et al., 2013). It is quite difficult to find studies of pregnant women with a larger sample size, especially a nationally representative sample. Using the Poisson regression option in G*power to conduct a *post hoc* power analysis, a sample size of 1,156 would give us >80% power to detect significant iron deficiency prevalence ratios of 1.5-2.0 (50-100% difference). However, we did not have enough power to detect the small observed differences between groups, especially with the low number of observed cases of anemia and IDA. We have added a description of the power calculation in the Methods (lines 91-96), Results (lines 240-251), and Strengths and Limitations (lines 360-363).

In our reporting, we followed the CDC guidelines (reference 47), which indicate that the effective number of cases should not be smaller than 30. We flagged such findings in our results. For instance, in Table 2, we indicate in the footnote that estimates from anemia and iron deficiency anemia in association with BMI categories may be unreliable due to a total number of cases less than 30.

Results:
It is better to put one figure to show the principle finding to make it easily understandable by the general reader.

Author comments: Thank you for this comment – we agree that figures are important and help the reader easily understand the findings. In this case, we present the continuous biomarker results, the first part of our main findings, in a figure. However, we felt that the details of our main prevalence model results were best displayed in a table. While we could add another figure to the paper, we were following the general guidelines of not replicating the same results across tables and figures.

Discussion:
Inflammation is predicted to be responsible for anemia among obese/overweight individuals, and CRP is a marker of inflammation. The author reported prevalence of women with CRP>0.5 mg/L. It was better to show the prevalence of women with iron deficiency based on the category of CRP.

Author comments: We welcome this valuable suggestion. We tested to see if inflammation (CRP>5 mg/L) was associated with cases of iron deficiency or IDA. We observed no differences by inflammatory status. This has been added to Table 1. Additionally, a prior publication by Mei et al., (reference #9) tested if inflammation impacts the prevalence of iron deficiency and anemia in pregnant women (used the same cycles as us but included a slightly different age range). Specifically, Mei et al., tested the results in the total sample and a subsample (removed pregnant women with CRP>5 mg/L) and found that the prevalence of ID in the subsample was not substantially different from the one from the total sample (reference #9). As such, our results are consistent with this prior publication.
