# Supplementary material for: Iron status and anemia in a representative sample of US pregnant women is not associated with pre-pregnancy BMI: Results from the NHANES (1999–2010) study
Source: PLoS One. 2024 Sep 10;19(9):e0300912. doi: 10.1371/journal.pone.0300912 (PMC11386453; doi:10.1371/journal.pone.0300912)
Supplement: Attachment — Submitted filename: Response to reviewers_May.29.2024.docx [file pone.0300912.s003.docx]

**Thank you to the editor for thoughtful consideration of our manuscript. Below are our responses to the comments from the editor in response to our last revision. Author responses are below editor comments in blue. All line numbers refer to the clean manuscript version.**

**Additional Editor Comments:**

The authors have made a good revision of the manuscript. However, I suggest reviewing and providing details on how the covariates were selected for multiple models to avoid residual confounding. The authors have replied that "We selected covariables a priori based on prior literature of factors known to be related to iron and anemia status and BMI during pregnancy (please see the Methods/Covariates section, lines 136-140)." - saying just "based on prior literature" is not enough! There are many ways to test multiple adjustment models. I recommend the use of a hierarchical conceptual framework combined with different p-values for each determinant level. Please see the following recent paper: NCD Risk Factor Collaboration (NCD-RisC). Worldwide trends in underweight and obesity from 1990 to 2022: a pooled analysis of 3663 population-representative studies with 222 million children, adolescents, and adults. Lancet. 2024 Mar 16;403(10431):1027-1050. doi: 10.1016/S0140-6736(23)02750-2.

**Author comments**: Thank you for your comments and explanation. We have carefully reviewed the request and would like to note that there are significant differences between our methods and the paper that the editor kindly shared (doi: 10.1016/S0140-6736(23)02750-2). In the shared paper, researchers conducted a multi-level, longitudinal analysis using Bayesian models. Our analysis used a cross-sectional design (not hierarchical or multi-level) of a large dataset using complex survey design to obtain a nationally representative sample in the US (DHHS publication; nos. (PHS) 2013–1360 & (PHS) 2012–1355; https://wwwn.cdc.gov/nchs/nhanes/analyticguidelines.aspx#sample-design). The weighting procedures required for complex survey designs make it difficult to assess true correlation and collinearity between covariates within a model. We reviewed current practices for covariate identification and adjustment and see others in the field apply a similar approach to ours. Recent articles in PLOS One have used similar approaches to ours when conducting a similar analysis (e.g. doi: 10.1371/journal.pone.0303169). Additionally, other analyses of iron status with the NHANES data used the same or similar covariates (see: doi: 10.3945/ajcn.110.007195, 10.1371/journal.pone.0112216, and 0.3945/ajcn.117.155978). Therefore, we feel that our analysis is most comparable to these previously published reports when we include a similar list of covariates. For these reasons, we consider that our method of covariate selection was the appropriate approach in this case. We acknowledge that using hierarchical Bayesian criteria may be preferred when a dataset has a hierarchical or multi-level structure. We have updated our Methods section to better describe our approach and reference comparable literature in lines 136-139.
